# Antiplatelet therapy contributes to a higher risk of traumatic intracranial hemorrhage compared to anticoagulation therapy in ground-level falls: a single-center retrospective study

**DOI:** 10.1007/s00068-022-02016-8

**Published:** 2022-06-22

**Authors:** Tomas Vedin, Jakob Lundager Forberg, Ebba Anefjäll, Riikka Lehtinen, Mohammed Faisal, Marcus Edelhamre

**Affiliations:** 1 Clinical Sciences, Lund University, Svartbrödragränden 3-5, 251 87 Helsingborg, Sweden; 2 Surgical Oncology Unit, Department of Surgery, Faculty of Medicine, Suez Canal University Hospital, Sheikh Zayed, Egypt

**Keywords:** (MESH), Brain injuries, Traumatic, Intracranial hemorrhages, Traumatic, Anticoagulants, Tomography, X-ray computed

## Abstract

**Background:**

Traumatic brain injury (TBI) is a common injury and constitutes up to 3% of emergency department (ED) visits. Current studies show that TBI is most commonly inflicted in older patients after ground-level falls. These patients often take medications affecting coagulation such as anticoagulants or antiplatelet drugs. Guidelines for ED TBI-management assume that anticoagulation therapy (ACT) confers a higher risk of traumatic intracranial hemorrhage (TICH) than antiplatelet therapy (APT). However, recent studies have challenged this. This study aimed to evaluate if oral anticoagulation and platelet inhibitors affected rate of TICH in head-trauma patients with ground-level falls.

**Methods:**

This was a retrospective review of medical records during January 1, 2017 to December 31, 2017 and January 1 2020 to December 31, 2020 of all patients seeking ED care because of head-trauma. Patients ≥ 18 years with ground-level falls were included.

**Results:**

The study included 1938 head-trauma patients with ground-level falls. Median age of patients with TICH was 81 years. The RR for TICH in APT-patients compared to patients without medication affecting coagulation was 1.72 (*p* = 0.01) (95% Confidence Interval (CI) 1.13–2.60) and 1.08 (*p *= 0.73), (95% CI 0.70–1.67) in ACT-patients. APT was independently associated with TICH in regression analysis (OR 1.59 (95% CI 1.02–2.49), *p* = 0.041).

**Conclusion:**

This study adds to the growing evidence that APT-patients with ground-level falls might have as high or higher risk of TICH than ACT-patients. This is not addressed in the current guidelines which may need to be updated. We therefore recommend broad prospective studies.

## Introduction

Traumatic brain injury (TBI) is a substantial cause of morbidity, mortality, and emergency department (ED) visits. Approximately 3% of all ED patients’ state isolated TBI as their chief complaint [1]. Correctly diagnosing TBI might be challenging, because initial signs and symptoms are not always indicative of the severity of the injury [2, 3]. Computerized tomography (CT) scan of the head is the gold standard for diagnosing TBI with almost perfect sensitivity but drawbacks such as a relatively high cost, prolongation of the ED visit and ionizing radiation with theoretical potential to cause head-and-neck malignancies [4–7]. Around 5–10% of TBI patients at the ED are diagnosed with traumatic intracranial hemorrhage (TICH) [8].

Epidemiology and medication among TBI patients have changed over the past 2 decades. In recent years, TBI patients are older when they are injured and the main trauma mechanism has shifted from motor vehicle accidents to falls [9]. Older studies reported the lowest incidence of TBI at 55 years, but conversely, newer studies report the highest incidence at 60 years [1, 8]. As we grow older, severity and number comorbidities increase as well as use of pharmaceuticals.

Anticoagulation therapy (ACT) and antiplatelet therapy (APT) are becoming more widespread. ACT has traditionally been considered to increase risk of TICH more than APT [10–12]. However, more recent studies have challenged this fact by reporting more TICHs in patients on APT than in patients on ACT [1, 13, 14]. The prevalence of TICH in patients with APT has been reported between 10 and 40% compared to 5 and 20% in ACT-patients. TICH seems to be lower in patients on direct-acting oral anticoagulants (DOACs) than in patients taking Vitamin-K Antagonists (VKAs) [1, 15–20].

This increased risk of TICH in ACT-patients is reflected in current TBI guidelines such as New Orleans Criteria (NOC), the National Institute for Health and Care Excellence (NICE) guidelines, the Canadian CT Head Rule (CCHR), Eastern Association for the Surgery of Trauma (EAST) practice management guidelines, and the Scandinavian Neurotrauma Committee Guidelines (SNC) by a head-CT recommendation [10–12, 21, 22]. There is ongoing debate regarding this risk, and to increase patient safety, SNC and EAST also recommend hospitalization regardless of CT-scan results to detect delayed intracranial hemorrhage [12, 22]. However, the potentially elevated risks for TICH and delayed intracranial hemorrhage in APT-patients are not similarly reflected in the guidelines. Only the SNC guidelines recommend special management for APT-patients (Head-CT to patients ≥ 65 years on APT) [12].

The CCHR is the only guideline that uses trauma energy level in the decision matrix by recommending a head-CT to patients who sustain medium- and high-level traumas. We have previously reported that trauma energy level could be explored as a potential rule-out criterion in otherwise healthy patients < 60 years [1]. Fakhry et al. [23] reviewed over 33.000 geriatric patients with ground-level falls in a trauma registry. They concluded that neither DOAC-therapy nor APT conferred an elevated risk of TICH except for an elevated incidence of TICH in patients on aspirin–clopidogrel combination therapy [23].

The rationale for performing this study was that it is feasible to assume that the changes in epidemiology and medication may have changed the spectrum of TBI patients that are at risk of developing TICH. If this holds to be true, this study could add to the body of evidence that is necessary to update current guidelines for management of TBI.

The aim of this study was to evaluate if any form of oral anticoagulation and platelet inhibitors affected rate of traumatic intracranial hemorrhage in head-trauma patients with ground-level falls.

## Materials and methods

Retrospective data from medical records on patients with isolated head trauma presenting at the ED at Helsingborg General Hospital were collected. The catchment area of 275,000 people generates 70,000 ED visits per annum. Tertiary neurosurgical care is provided at Skåne University Hospital in Lund. This hospital is separate from Helsingborg General Hospital and located 40 km away. The SNC guideline for TBI was used as institutional guideline during the entire study period [12].

Data collection was done between January 1, 2017 to December 31, 2017 and January 1, 2020 to December 31, 2020. Database was collected as part of a more comprehensive research project, and the gap between 2017 and 2020 was not pertinent to the present article.

The inclusion criteria were

- Registration in the ED information system as “head trauma” (as triaged by an emergency department nurse)
- Age ≥ 18 years
- Ground-level fall.

Exclusion criteria were

- Scheduled ED return-visits
- Duplicate records (two medical records with the same information)
- Empty medical records (record entries without any information)
- Visits managed by a nurse without physician’s involvement
- Too trivial visits (e.g., trauma to the head without trauma to the neurocranium and a small cut in need of stitches)
- Classified medical records (records with security restrictions we could not access)
- Trauma-level unknown or other than ground-level fall.

The following parameters were collected and analyzed in the present study:

1. Age (years)
2. Gender (male/female)
3. Age-Adjusted Charlson Comorbidity Index
4. Head CT performed (yes/no)
5. Head-CT outcome (hemorrhage/no hemorrhage)
6. Admission to general hospital ward (yes/no)
7. Admission to intensive-care unit or neurointensive care unit (yes/no)
8. Neurosurgical intervention (yes/no)
9. Level of consciousness using Reaction Level Scale 85 (1–8)
10. Past illnesses (yes/no)
11. Anticoagulant treatment (no/warfarin/novel anticoagulant/low-molecular-weight heparin)
12. Platelet inhibitor treatment (no/acetylsalicylic acid/clopidogrel/ticagrelor/prasugrel/dipyramidol/combinations)
13. Other medication (yes/no)
14. New focal neurological deficits (yes/no)
15. Nausea (yes/no)
16. Vomiting (yes/no)
17. Amnesia, type, and duration (yes/no, antegrade/retrograde, time hh:mm)
18. Loss of consciousness (yes/no)
19. Peritraumatic seizure (yes/no)
20. Trauma mechanism

One patient could be included several times in the study if there was a new trauma.

Primary outcome measures for study aim were risk ratio for TICH and difference in rate of TICHs in patients on ACT and APT. Secondary outcome measure was odds ratio for association with TICH.

Comorbidity and age were quantified with the age-adjusted Charlson Comorbidity index (aa-CCI) which is validated for both prospective and retrospective use [26–29].

All hospitals in the Skåne Region share the same electronic medical record system. Examined medical records included ED records, medical records from the ward (if admitted), laboratory results, and radiology reports from the entire Skåne Region. Medical records up to 365 days before the ED visit from the entire Skåne Region were reviewed for current medication and other comorbidities not stated in the ED records. In cases of missing data in the physician’s records, other staff’s (e.g., nurses, paramedics etc.) notes were reviewed. Records were also reviewed 6 months after index visit to ensure that no TICHS were missed, but the total number of ED visits per patient during this follow-up period was not recorded.

Data were collected by three reviewers. To minimize information bias, guidelines for retrospective medical record review by Vassar and Holzman were followed [30]. This meant creating and following a comprehensive pro forma document which stated how different situations should be interpreted and coded and stating clear inclusion and exclusion criteria prior to information collection. To further enhance information reliability, a Cohen’s kappa analysis of 100 randomized medical records reviewed by two researchers was done. All reviewed parameters but new neurological deficits and LMWH-treatment had good or very good agreement. Please see previously published study for more details [16].

## Data definitions and missing data

TICH was defined as intracranial hemorrhage diagnosed by head-CT scan at index ED visit. Absence of TICH was defined as not finding TICH on index CT or treating physician not prescribing a CT. Intervention was defined as surgical intervention, intubation, or intensive-care of any sort because of the head trauma. Death due to TBI was defined as death because of the head injury that occurred during the hospital stay. APT was defined as any or several simultaneous drugs affecting thrombocyte function and no concurrent ACT. ACT was defined as any drug affecting coagulation factors and no concurrent APT. Ground-level trauma was defined as fall from standing or sitting.

Missing data in all parameters except level of consciousness was coded as such but handled pragmatically in analysis by interpreting it as the absence of pathology (e.g., no mention of loss of consciousness was analyzed as “no loss of consciousness”). This choice was based on our experience that ED-physicians usually write pragmatic medical records and report positive findings but not negative findings. The extracted parameters are routinely assessed when caring for TBI patients and it was therefore considered as an acceptable risk of systematic information bias. Missing level of consciousness was handled as missing in descriptive analysis as well as in regression analysis.

To make the study more internationally eligible, level of consciousness is reported throughout the study as GCS. In Sweden, Reaction Level Scale (RLS) is more commonly used than GCS [24]. The GCS of the present study was obtained from the RLS-number from physician’s medical records, either by stated RLS-number or by interpreting the degree of consciousness from the text in the medical records (e.g., patient is wide awake = RLS1). In cases where physician’s notes were insufficient, triage nurse’s or paramedics’ notes were used. Since it previously has been shown that RLS correctly classifies GCS 15 and 14, but that there are discrepancies when converting between RLS-3-8 and GCS13-3, level of consciousness was converted to GCS but to avoid bias, only reported as GCS15-14 and GCS < 14 [25].

## Statistical analysis

Data were analyzed with SPSS version 27 for Mac. Shapiro–Wilk formula and histograms were used to investigate data distribution. Non-parametric data were presented with median, and 25th and 75th percentiles (Q1 and Q3). TICH-prevalence in ACT- and APT-cohorts was compared to TICH-prevalence in patients without these medications with χ^2^ test. Risk ratios for TICH with 95% confidence intervals (CIs) were calculated for the ACT- and APT-groups compared to all patients with ground-level falls. *P* < 0.05 was considered statistically significant except in first step of regression analysis.

Binary logistic regression was performed to assess if results of χ^2^-analysis and risk ratio calculation were due to confounding or actual association in a multivariate analysis. Features commonly associated with TICH, aa-CCI and ACT/APT were used as covariates. Aa-CCI was subdivided pragmatically according to odds ratio (OR) and CIs. The regression was performed in three steps where the first step was a univariate logistic regression with TICH as dependent variable. In the second step, independent variables with *p* < 0.4 from the first step were included in a multiple logistic regression. The third step was performed as a multiple logistic regression with all the independent variables that had *p* < 0.05 in the second step. In all steps, p value and OR with 95% CI were reported. Nagelkerke R^2^ was reported as determination coefficient. Potential multicollinearity between independent variables was examined with correlation matrix where correlations below or above ± 0.6 were considered acceptable multicollinearity for inclusion in multiple regression analysis.

## Results

**Fig. 1:**
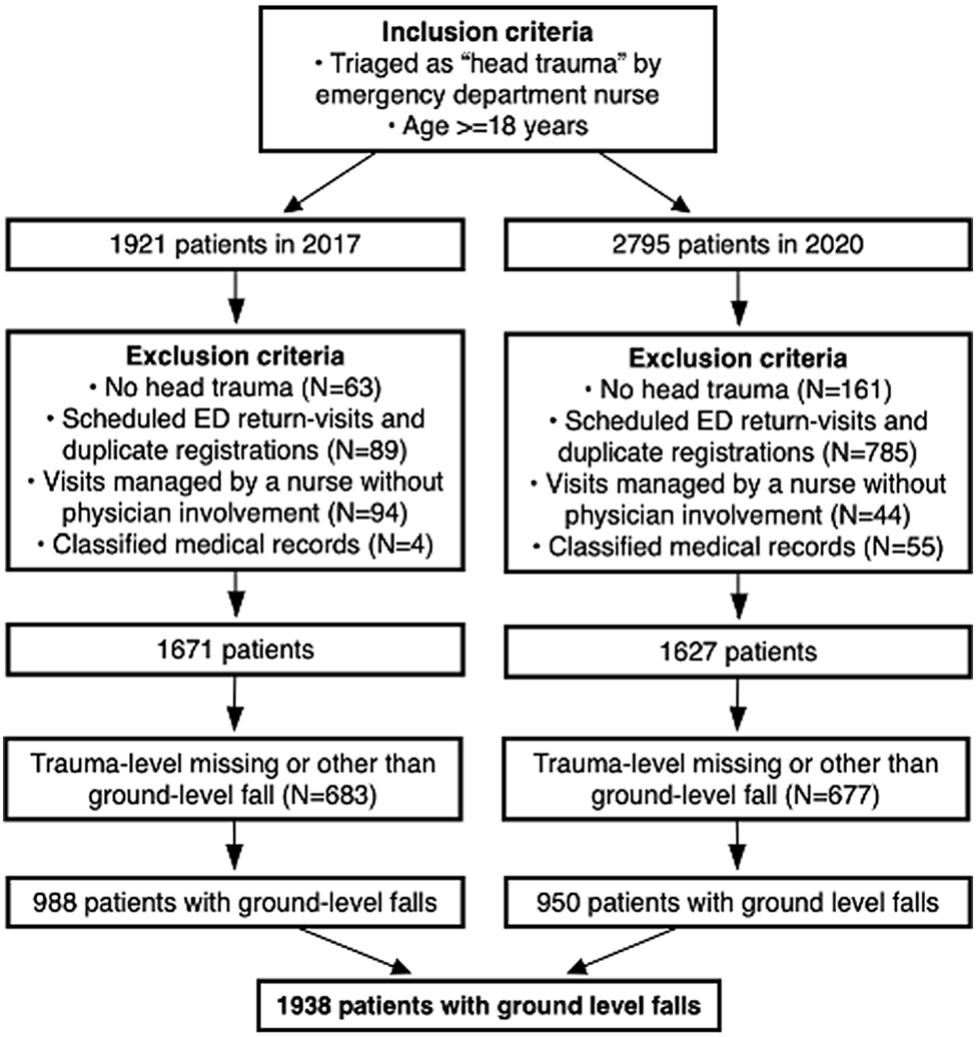
Inclusion process of the entire study with 988 included patients from 2017 and 950 included patients from 2020, all with ground-level falls

A total of 1938 head-trauma patients had ground-level falls (988 in 2017 and 950 in 2020) during the inclusion period. See Fig. 1 for inclusion process.

TICH was diagnosed in 120 patients and 60 of these were treated with either ACT or APT. TICH was found in 5.4% of patients not on medication affecting coagulation, 5.8% of ACT-patients and 9.2% of APT-patients had TICH. A total of 1/14 (7.1%) patients had TICH in the LMWH-treatment subgroup and 1/14 (7.1%) patients had TICH in subgroup with combinations of ACT and APT.

Median age for all patients with TICH was 81 (73, 88) years. See Table 1 for general data on patients with ground-level falls with and without ACT or APT.

**Table 1:** Characteristics of head-trauma patients because of ground-level falls

Variable name	Ground-level falls			
	All patients	Patients without anticoagulation or antiplatelet therapy	Anticoagulantion therapy	Antiplatelet therapy
	(*n* = 1938)	(*n* = 1080)	(*n* = 483)	(*n* = 347)
Age [median (Quartile 1, Quartile 3)] years	77 (64, 86)	69 (52, 81)	85 (77, 89)	81 (74, 88)
Gender				
Male	864 (44.6%)	471 (43.6%)	217 (44.9%)	165 (47.6%)
Female	1074 (55.4%)	609 (56.4%)	266 (55.1%)	182 (52.4%)
Glasgow Coma scale (GCS)^*^				
GCS 14–15	1891 (97.6%)	1047 (98.8%)	475 (99.4%)	342(99.2%)
GCS < 14	18 (0.9%)	13 (1.2%)	3 (0.6%)	1 (0.3%)
Patient history				
Vomiting	115 (5.9%)	85 (7.9%)	13 (2.7%)	17 (4.9%)
Loss of consciousness	523 (27.0%)	331 (30.6%)	97 (20.1%)	90 (25.9%)
Amnesia	407 (21.0%)	263 (24.4%)	68 (14.1%)	71 (20.5%)
Age-adjusted Charlson comorbidity index				
≤ 8	1880 (97.0%)	1067 (98.8%)	455 (94.2%)	335 (96.5%)
> 8	58 (3.0%)	13 (1.2%)	28 (5.8%)	12 (3.5%)
Clinical findings, outcomes, and interventions				
New focal neurological deficits	110 (5.7%)	62 (5.7%)	22 (4.6%)	26 (7.5%)
Peritraumatic seizures	29 (1.5%)	21 (1.9%)	2 (0.4%)	6 (1.7%)
Traumatic intracranial hemorrhage	120 (6.2%)	58 (5.4%)	28 (5.8%)	32 (9.2%)
Head computerized tomography performed	1457 (75.2%)	660 (61.1%)	461 (95.4%)	310 (89.3%)
Admission to hospital	720 (37.2%)	263 (24.4%)	309 (64%)	129 (37.2%)
Intervention^**^ during index visit	8 (0.4%)	5 (0.5%)	2 (0.4%)	1 (0.3%)
Death due to head injury	13 (0.7%)	9 (0.8%)	2 (0.4%)	1 (0.3%)

^*^Variable Glasgow Coma Scale has 29 missing cases

^**^Neurosurgical operation, intubation, or care at intensive-care unit because of head injury. The 14 cases with low-molecular-weight heparin treatment and the 14 cases with combinations of anticoagulation and antiplatelet therapy are not included in table

APT was found in 347 (17.9%) of all patients with ground-level falls and acetylsalicylic acid (ASA) was the most common drug class found in 253 (72.9%) of the APT-patients. ACT was found in 483 (24.9%) of all patients with ground-level falls. DOAC was the most common ACT found in 294 (60.9%) patients and VKAs were found in 161 (33.3%) patients. See Table 2 for complete distribution of TICHs and different medications affecting coagulation.

**Table 2:** Distribution of drugs affecting coagulation and prevalence of traumatic intracranial hemorrhage with ground-level falls

Type of therapy	Type of medication	No intracranial hemorrhage (%^*^)	Intracranial hemorrhages (%^**^)
Antiplatelet therapy	Acetylsalicylic acid 75 mg	253 (72.9%)	26 (10.3%)
	Clopidogrel	47 (13.5%)	4 (8.5%)
	Cilostazol 100 mg	10 (2.9%)	0 (0.0%)
	Dual antiplatelet therapy	5 (1.4%)	2 (40.0%)
	Warfarin	161 (33.3%)	8 (5.0%)
Anticoagulation therapy	Apixaban	228 (47.2%)	15 (6.6%)
	Dabigatranexilat	11 (2.3%)	1 (9.1%)
	Edoxaban	53 (11.0%)	4 (7.6%)
	Rivaroxaban	2 (0.4%)	0 (0.0%)
Low-molecular-weight heparin therapy	***	13 (92.9%)	1 (7.1%)
Combination of antiplatelet and anticoagulation therapy	****	13 (92.9%)	1 (7.1%)
No antiplatelet or anticoagulation therapy	–	1022 (94.7%)	58 (5.7%)

Table showing distribution of patients on different drugs within the drug classes “antiplatelets” and “anticoagulants”

^*^Percentage of patients within entire drug class

^**^Percentage of patients within specific subtype of antiplatelet or anticoagulation drug

^***^Either single therapy of tinzaparin or enoxaparin

^****^Combinations of acetylsalicylic acid, clopidogrel, ticagrelor, or prasugrel and warfarin, apixaban, dabigatran, or edoxaban

The RR for TICH in APT-patients compared to patients without medication affecting coagulation was 1.72 (*p* = 0.01) (95% CI 1.13–2.60) and 1.08 (*p* = 0.73), (95% CI 0.70–1.67) in ACT-patients.

The rate of TICH in patients on APT (32/347) was significant compared to the rate of TICH in patients without APT, ACT, or combinations thereof according to the χ^2^ test (*p* = 0.01).

The rate of TICH in patients on ACT (28/483), *p* = 0.73) was not statistically significant compared to the rate of TICH in patients without APT, ACT, or combinations thereof according to the χ^2^ test.

See Table 3 for logistic regression analysis of factors commonly associated with TICH.

**Table 3:** Logistic regression of variables affecting intracranial hemorrhage with traumatic intracranial hemorrhage as dependant variable

Variable	Univariate regression		Multiple regression 1		Multiple regression 2	
	*P*	OR^3^ (95% CI^4^)	P	OR^3^ (95% CI^4^)	*P*	OR^3^ (95% CI^4^)
aaCCI^1^ > 8	0.065	2.15 (0.95–4.84)	0.046	2.38 (1.01–5.60)	0.046	2.38 (1.02–5.57)
GCS^2^ < 14	< 0.001	10.21 (3.89–26.83)	0.004	4.93 (1.63–14.96)	0.001	5.97 (2.01–17.35)
Signs of basal skull fracture	0.115	2.15 (0.83–5.59)	0.843	1.00 (0.99–1.01)	–	–
Signs of depressed fracture	0.796	0.90 (0.38–2.10)	–	–	–	–
Loss of consciousness	0.001	1.89 (1.29–2.76)	0.087	1.46 (0.95–2.25)	–	–
Posttraumatic vomiting	0.126	1.67 (0.87–3.19)	0.795	1.06 (0.54–2.26)	–	–
Posttraumatic seizures	0.019	3.25 (1.22–8.68)	0.425	1.58 (0.51–4.89)	–	–
New focal neurological deficits	< 0.001	5.71 (3.51–9.29)	< 0.001	4.30 (2.51–7.35)	< 0.001	4.48 (2.64–7.59)
Amnesia	0.013	1.67 (1.11–2.51)	0.049	1.59 (1.02–2.50)	0.004	1.85 (1.22–2.83)
Sex	0.635	0.91 (0.63–1.32)	–	–	–	–
Anticoagulation therapy	0.678	0.91 (0.59–1.41)	–	–	–	–
Antiplatelet therapy	0.011	1.74 (1.14–2.65)	0.041	1.60 (1.02–2.50)	0.041	1.59 (1.02–2.49)

Table shows regression analysis of variables affecting odds of traumatic intracranial hemorrhage in three steps with eight variables left in the final regression model. Determination coefficient *R*^*2*^ = 0.085. Correlation matrix showed values between −0.540 and 0.331

1—Age-adjusted Charlson Comorbidity index. 2—Glasgow coma Scale. 3—Odds Ratio. 4—Confidence interval

## Discussion

The present study describes occurrence of TICH in TBI patients with ground-level falls. A large cohort of 1938 patients with 347 patients on APT and 483 patients on ACT was analyzed. The results showed that treatment with APT was independently associated with TICH. However, the same association was not found between TICH and ACT. The results indicate that the risk of TICH in patients treated with ACT could be as low as for patients not taking any ACT or APT. Furthermore, characteristics generally associated with elevated risk of TICH (e.g., vomiting and loss of consciousness, etc.) were not associated with TICH after ground-level falls in the present analysis.

The significantly higher rate of TICH in APT-patients compared to TICH-rate in ACT-patients was contradicted by the overlapping RR 95% CIs. This could be due to insufficient sample size. To explore if there was an actual association between TICH-incidence based on ACT or APT, a regression analysis was performed. It was prudent to assume that ACT and/or APT could be surrogate markers for other potential risk factors such as frailty and high age or that they were just confounders that covaried with important symptoms and signs such as loss of consciousness, amnesia, etc. However, regression analysis showed that treatment with APT entailed elevated odds for TICH, and that this odds was independent from age and comorbidity quantified by the aa-CCI. Furthermore, treatment with ACT had no association with outcome of intracranial hemorrhage. Therefore, it is feasible that there is an actual difference in TICH-incidence between patients treated with ACT and APT and that a larger sample size would render non-overlapping CIs for the RR. The higher risk for TICH in APT-patients is not reflected in all the current guidelines and could potentially mean that some TICHS could be missed [10–12, 21].

Signs, symptoms, and items from the patient history such as post-TBI vomiting, loss of consciousness, amnesia, and seizures are traditionally regarded as important when risk-stratifying patients with TBI [10, 12, 21]. However, only amnesia was associated with TICH in the final regression model. It is difficult to know from the present study how much the sample size affects this analysis, but it can be assumed that these factors are not as important when risk-stratifying patients with ground-level trauma. A possible explanation for this could be that the patients that sustain ground-level falls, because of high age and frailty, are more sensitive to this relatively small trauma energy and thus more prone to develop TICH. However, the trauma energy might not be high enough to cause a concussion and subsequent concussion symptoms such as vomiting and loss of consciousness. These symptoms can also appear because of a TICH, but in this scenario, they typically appear hours after the trauma as meningism arises and the intracranial pressure rises and might not be present when the patient presents to the ED. Consequently, a guideline recommendation based on these features might erroneously risk-stratify some patients with ground-level falls, because the features could be absent, even if the patient has developed a TICH.

The median age of patients in this study is higher than ED cohorts that include TBI patients with all trauma levels [8, 16, 31]. The reason for this could be that ground-level falls that lead to a concussion in younger individuals are less common and do not cause injuries severe enough to prompt an ED visit. Despite this age difference, the rate of TICH in the entire cohort was around the same level as most other ED cohort studies [8]. Age of 60–65 years is an absolute CT-indication in most of the large guidelines such as CCHR, NOC and NICE, whereas the SNC guidelines recommend a CT to all patients ≥ 65 years with APT [10–12, 21]. The SNC recommendation is more in line with the occurrence of TICH after ground-level falls, at least based on the results of the present study. A mandatory CT of TBI patients over 60 years might not be necessary and study of risk stratification based on APT among other features could theoretically help reduce the number of CTs.

It is also possible that a study looking at sub-classes or combinations of APT/ACT would indicate an elevated risk of certain pharmaceuticals (e.g., dual APT or some types of DOACs). The distribution of TICH in the present study within the two drug classes and in combination of APT and ACT could indicate this, but the numbers were deemed too small for further statistical analysis. Fakhry et al. [23] found that the incidence of TICH was not increased because of ASA single therapy [23]. Moreover, van den Brand et al. [32] (meta-analysis of 20, 247 patients) concluded the same in patients with mild TBI (GCS 14–15 upon index visit) [32]. However, both studies found increased risk in patients on aspirin–clopidogrel combination therapy [23, 32]. The conclusions of these two studies differ from the present study, but high-level evidence is still lacking, and to remedy this, we recommend prospective studies.

The present study’s relatively low incidence of TICH in ACT-patients has been reported by several other studies in the past years [1, 15–20]. Even though one-third of the ACT-patients in the present study were on VKAs and not DOACs, the TICH-incidence did not differ significantly from the TICH-incidence in the cohort of patients with ground-level traumas not on ACT or APT. Given the state of current research where VKA has shown higher TICH-incidence compared to DOAC, it is possible that the TICH-incidence would be even lower in this subgroup in a population where VKAs were less frequent [1, 15, 16]. If the RR of 1.78 for APT-patients can be properly demonstrated in a study with sufficient sample size, there is a substantial TICH-hazard for APT-patients. Furthermore, the increasing evidence showing that DOAC is safer than VKA is an indication that guidelines handling these patient categories differently are needed. A recent meta-analysis concluded that a selective use of head-CT in TBI patients with DOAC instead of a mandatory CT might be feasible [33].

The most important limitation of the present study is the retrospective data collection. This can lead to information bias both when interpreting medical records and handling missing data. Irrespective of counter-measures, this prevents us from drawing more than cautious conclusions from this study. The effect of the measures we took to reduce interpretation bias (e.g., following guidelines for retrospective reviews in general and the pro forma document in particular) was evident in the Cohen’s kappa coefficient analysis. It showed that the concordance between two separate reviewers was acceptable. Hence, data were collected in a fairly reproducible manner and this can be construed as increased data validity in the retrospective setting.

The pragmatic approach to missing data was discussed in detail in the research group prior to information gathering and deemed the best solution. Because it is not based on statistical methods, it bares the drawback of not giving any reliability measurement (e.g., confidence interval). Because of this, it is difficult to know the direction of this bias.

The choice to interpret “Head-CT not performed” as absence of TICH can lead to missed intracranial hemorrhages. However, it is unlikely that any TICHs with serious consequences were missed since screening for new ED visits 6 months after the index-TBI was performed. These events might have been missed if they took place outside the Region Skåne catchment area, but that risk was deemed small.

## Conclusion

This study adds to the growing evidence that APT-patients with ground-level falls might have as high or higher risk of TICH than ACT-patients. This is not addressed in the current guidelines which may need to be changed to be in line with the current head-trauma epidemiology and mechanism of injury. We therefore recommend prospective studies with a broad range of risk factors investigated.

## Data Availability

Data will be made available upon request.
